# A Case of Blunt Trauma-induced ST-elevation Myocardial Infarction Sustained During a Prison Brawl

**DOI:** 10.7759/cureus.6733

**Published:** 2020-01-22

**Authors:** Zachary T Clark, Natalie Thiel, John Perry, Michael R Minckler

**Affiliations:** 1 Molecular Biology, Whitman College, Walla Walla, USA; 2 Epidemiology and Public Health, University of Washington, Seattle, USA; 3 Interventional Cardiology, Providence St. Mary Medical Center, Walla Walla, USA; 4 Emergency Medicine, Providence St. Mary Medical Center, Walla Walla, USA

**Keywords:** traumatic myocardial infarction, stemi, traumatic plaque rupture, mi, prison, brawl

## Abstract

Myocardial infarction (MI) is a serious and time-sensitive condition. MIs are typically seen in patients with coronary artery disease (CAD) and are caused by the rupture of an atherosclerotic plaque due to factors contributing to plaque instability. However, this case illustrates that plaque rupture can also be caused by blunt trauma to the chest. Considering MI as a possible result of chest trauma may decrease time from presentation to diagnosis and treatment and, therefore, improve outcomes in similar cases, particularly when patients presents unusually or with very few risk factors for MI.

## Introduction

Acute myocardial infarction (AMI) from blunt cardiac trauma is an uncommon condition and is rarely mentioned in the medical literature [1]. Blunt cardiac trauma is known for instantly causing myocardial contusion, myocardial rupture or aortic injury. AMI is generally a result of chronic plaque buildup from coronary artery disease (CAD). Blunt cardiac trauma and AMI are therefore seldom connected in clinical settings. The most commonly recognized risk factors of AMI are increased age (≥45 years old), history of smoking, and patient history or family history of CAD [2]. This case report of ST-elevation myocardial infarction (STEMI) caused by left-anterior descending (LAD) coronary artery occlusion in a 30-year-old man with no known history of smoking and no history of CAD shows that AMI in patients with blunt cardiac trauma can occur without any of these risk factors. This case report demonstrates that AMI should be considered a possibility in instances of blunt cardiac trauma and that quick diagnosis of this uncommon condition is critical to successful patient outcomes.

## Case presentation

A 30-year-old male patient presenting to the emergency room (ER) sustained a jaw laceration and mid mandible pain, following involvement in a penitentiary brawl. His known medical history was limited to prior methamphetamine use. His vital signs included blood pressure 134/98, heart rate of 84 bpm, respiratory rate of 21, and SpO2 of 100%. During the brawl, he received several blows to the chest, head, and neck, including a kick to the anterior chest wall. He sustained the jaw laceration when thrown to the ground.

**Figure 1:**
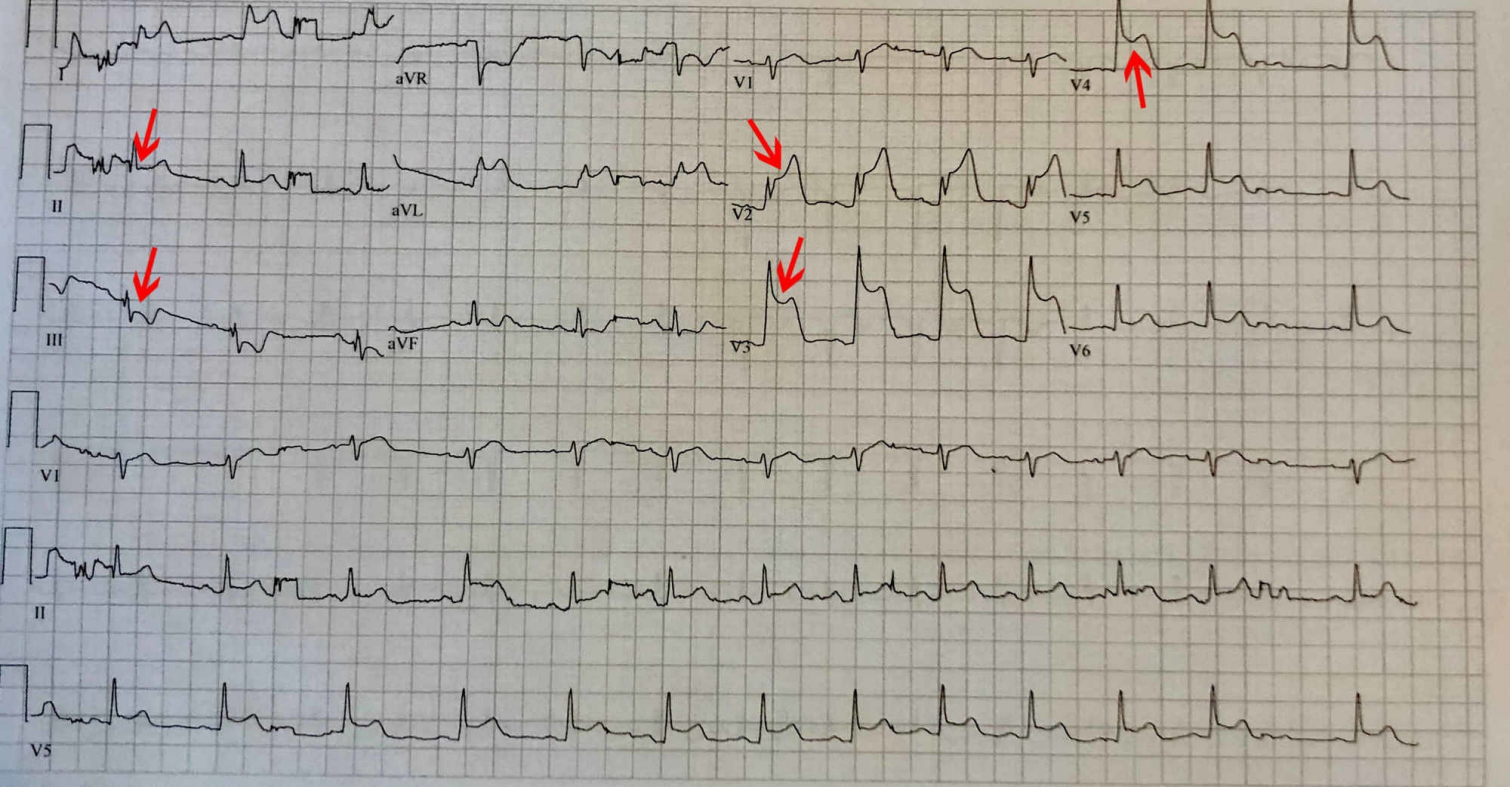
Electrocardiogram (EKG) showing 5-7mm of ST elevation in the anterior precordial leads and reciprocal ST depression in limb leads indicative of ST-elevation myocardial infarction (STEMI)

After arrival at the ER, he reported the onset of chest pain which continued to worsen. An electrocardiogram (EKG) showed anterolateral ST-elevation myocardial infarction (STEMI), therefore, a STEMI alert was paged out (Figure 1). While on the monitor, he developed ventricular tachycardia with brief syncope. The arrhythmia spontaneously resolved before going into spontaneous ventricular fibrillation arrest, which lasted approximately five seconds. The arrhythmia spontaneously resolved again. IV access was established at each antecubital fossa. A normal saline bolus and amiodarone bolus were administered. A bedside echocardiogram showed anterior wall motion abnormality. Due to the significant risk for hemodynamic instability, he was intubated and transferred to the cardiac catheterization laboratory (cath lab). A post-intubation chest X-ray was obtained showing a normal cardiac silhouette and clear lungs.

**Figure 2:**
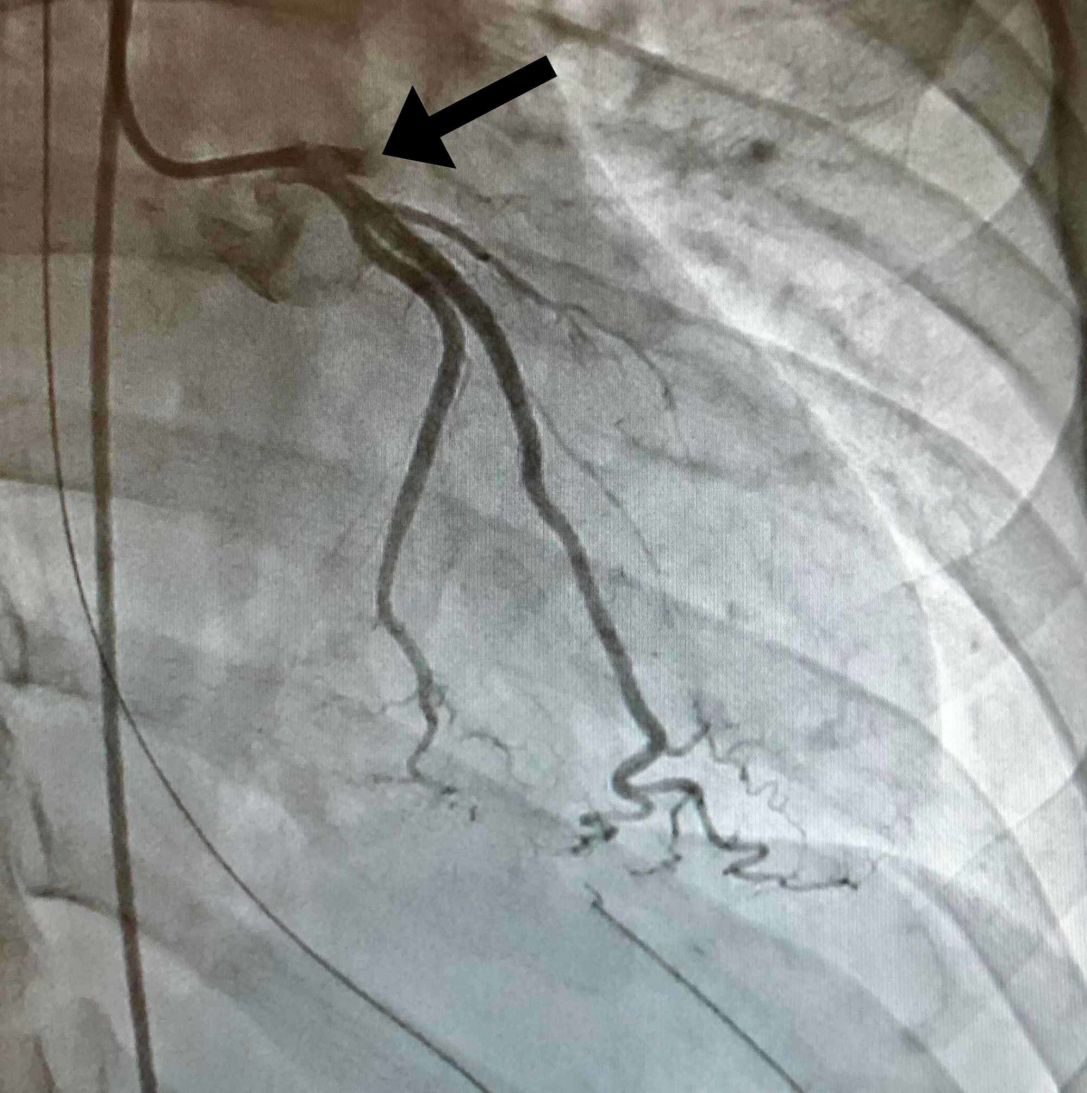
Total occlusion of the left-anterior descending (LAD) artery at its origin before intervention

**Figure 3:**
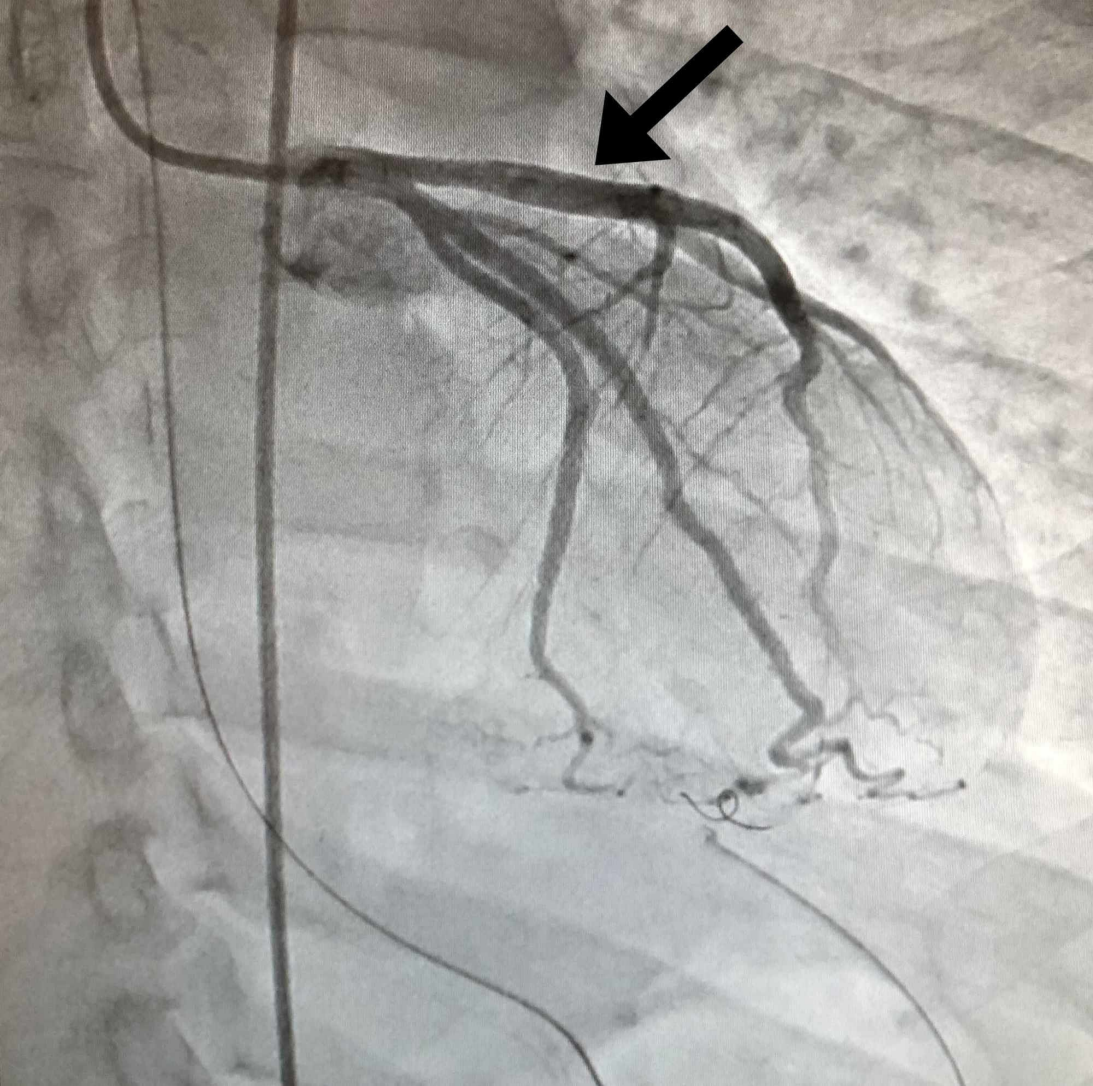
Normal flow restored after intervention

Initial labs drawn during the ER course including complete blood count, complete metabolic panel, troponin, and brain natriuretic peptide (BNP) showed no abnormality. Electrocardiography found a sinus rhythm with 5-7mm of ST elevation in the anterior precordial leads with reciprocal ST depression in limb leads. Thoracic aortography found normal ascending, transverse, and descending segments of the aorta. No significant aortic regurgitation or evidence of dissection was present. Left ventriculography revealed the left ventricle was of normal volume. The anterobasal, anterolateral, and apical segments were akinetic and the mid inferior segment was hypokinetic. Left ventricular ejection fraction was found to be 20%. Coronary angiography revealed total occlusion of his left-anterior descending artery (LAD) at its origin (Figure 2). Near the origin, homogenous plaque or hemorrhage into a plaque was apparent with an area of stenosis greater than 70%. His LAD was found to be 5.5 mm in diameter at the origin and 5 mm in diameter near the origin of the major diagonal branch per intravascular ultrasound. The occlusion was resolved with mechanical thrombectomy, vigorous antiplatelet therapy, anticoagulant, and stenting (Figure 3). Following the procedure, he was started on a statin, aspirin, Plavix, an angiotensin-converting enzyme (ACE) inhibitor, and a beta blocker.

His jaw laceration was repaired with 4 staples. Suspected aspiration pneumonitis following his syncopal episodes was treated with piperacillin/tazobactam followed by amoxicillin/clavulanic acid. Five days later, he had a sudden onset of left upper quadrant pain. A computed tomography scan confirmed a splenic infarct involving approximately 25% of his spleen. Etiology for this splenic infarct is unknown but embolic phenomenon was suspected.

## Discussion

Blunt force trauma very rarely causes MI or cardiac damage [3]. In the few cases where blunt trauma does result in cardiac injury, the LAD coronary artery is the most commonly injured vessel as is the case of our patient [4]. Notably, this case is rare as, other than remote methamphetamine abuse, the patient did not have the usual risk factors for plaque formation or rupture and was significantly younger than reported cases [3]. 

Important to note from this case, clinical diagnosis for MI is challenging in the context of trauma due to atypical clinical presentation. While our patient did experience the onset of classical MI symptoms preceding rapid decompensation, his initial complaint was limited to his chin laceration. Reviews of trauma-induced MI have shown that patients rarely present with chest pain or pressure, pain from other injuries, or analgesics that have been administered and can have obscuring effects [1]. Timely diagnosis and aggressive management of MI is made particularly challenging by its varied clinical presentation. 

Traumatic MI poses important therapeutic dilemmas once identified. Contraindications for thrombolytic procedures include major trauma, hemorrhage, and suspected aortic dissection, which removes a majority of trauma patients. Examples of direct percutaneous coronary intervention following traumatic MI have been reported, but are exceedingly rare [5-6]. Surgical intervention may be the only recourse in complicated traumatic cases of MI [7]. While rare, it is important to consider a possible MI following trauma, especially as a cause for sudden, unexplained decompensation.

## Conclusions

AMI is a diagnosis to consider in any trauma patient who has experienced blunt chest wall trauma. Given the availability of electrocardiogram and troponin testing in the ER, the benefit of doing these minimally invasive tests vastly outweigh the risk of missing this diagnosis. More studies and reporting to the medical literature are needed to figure out if these injuries have more to do with precisely placed blunt trauma or if risk from such an injury is elevated due to methamphetamine abuse at any point in life. Most importantly, once the diagnosis of AMI secondary to blunt chest-wall trauma is made, the treatment pathway for this disease may be vastly different than AMI alone as trauma patients may have additional injuries that preclude them from receiving anticoagulants used commonly in cath lab therapy.
